# 809. Empiric Antifungal Therapy Is Not Associated with Improved Survival Outcome among Intensive Care Units (ICU) Patients

**DOI:** 10.1093/ofid/ofad500.854

**Published:** 2023-11-27

**Authors:** Lauren Wong, Dimitar Zelenkov, Maureen Campion, Kap Sum Foong, Shira Doron, Gabriela Andujar Vazquez

**Affiliations:** Tufts University, Boston, Massachusetts; Tufts Medical Center, Boston, Massachusetts; Tufts Medical Center, Boston, Massachusetts; Tuft Medical Center, Boston, Massachusetts; Tufts Medical Center, Boston, Massachusetts; Tufts Medical Center, Boston, Massachusetts

## Abstract

**Background:**

In recent years, ICU at Tufts Medical Center (TMC) have a higher utilization rate of antifungal agents compared to nationwide ICU data reported to the National Health and Safety Network. The role of empiric antifungal therapy in reducing ICU patient mortality remains controversial. Prudent use of antifungals is needed to mitigate the risk of further antifungal resistance development. Our study aimed to evaluate empiric antifungal use among ICU patients and its association with ICU 30-day all-cause mortality.

**Methods:**

We conducted a retrospective study of all adult ICU patients at TMC who had received fluconazole (F) or micafungin (M) between August 2022 and February 2023. We collected demographics, comorbidities, *Candida* score, indication for empiric antifungal therapy, use of mechanical ventilation (MV) and broad spectrum antibiotic use. Univariable and multivariable logistic regression analyses were conducted to identify predictors associated with ICU 30-day all-cause mortality among ICU patients who received antifungal therapy.

**Results:**

A total of 77 ICU patients received antifungal treatment, (F: 22 patients, M: 55 patients). The median treatment duration was the same for both therapies, 2 days [1-4]. Only 4 (5%) of patients had positive blood cultures for *Candida sp*. The median *Candida* score was 3 [2-4]. Antifungal therapy was initiated for suspected infection in 52 (68%) of patients per prescriber orders indications. The majority of patients were ventilated (63, 81.8%), of these, 15 (23.8%) were on extracorporeal membrane oxygenation. The 30-day all-cause mortality was 58 (75%). Multivariable logistic regression analysis showed antifungal therapy for ≥ 48 hours was not associated with improved ICU mortality (adjusted odds ratio, 0.276; 95% confidence interval, 0.31-2.44). Chronic heart failure was associated with an increased risk for ICU mortality (adjusted odds ratio, 19.91; 95% confidence interval, 1.32-299.72).

**Conclusion:**

A high 30-day all-cause mortality among ICU patients who received empiric antifungal treatment was observed. Similar to national trends, the incidence of fungemia was low despite moderate candida scores. Empiric antifungal therapy ≥48 hours was not associated with improved mortality outcome in this population.

**Disclosures:**

**Maureen Campion, PharmD, BCIDP**, Shinoigi: Speaker

